# The Integration of Occlusion and Disparity Information for Judging Depth in Autism Spectrum Disorder

**DOI:** 10.1007/s10803-017-3234-x

**Published:** 2017-07-07

**Authors:** Danielle Smith, Danielle Ropar, Harriet A. Allen

**Affiliations:** School of Psychology, University of Nottingham, Nottingham, NG7 2RD UK

**Keywords:** Autism spectrum disorder, Occlusion, Disparity, Cue integration, Depth, 3D

## Abstract

In autism spectrum disorder (ASD), atypical integration of visual depth cues may be due to flattened perceptual priors or selective fusion. The current study attempts to disentangle these explanations by psychophysically assessing within-modality integration of ordinal (occlusion) and metric (disparity) depth cues while accounting for sensitivity to stereoscopic information. Participants included 22 individuals with ASD and 23 typically developing matched controls. Although adults with ASD were found to have significantly poorer stereoacuity, they were still able to automatically integrate conflicting depth cues, lending support to the idea that priors are intact in ASD. However, dissimilarities in response speed variability between the ASD and TD groups suggests that there may be differences in the perceptual decision-making aspect of the task.

## Introduction

Although Autism Spectrum Disorders (ASD) are generally associated with social difficulties, recent changes to diagnostic criteria (i.e. DSM-V) have led to a renewed interest in the sensory symptoms within this population. One sensory domain which has commonly been reported to show distinct differences is visual perception. Specifically, it has been suggested that atypical integration of visual information may explain why individuals with ASD perform better (Bertone et al. 2005; O’Riordan et al. 2001) or worse (Bertone et al. 2003; Van Boxtel and Lu 2013) than typically developing individuals on some perceptual tasks.

## Theories of Perception in Autism

Currently, there is not a consensus on how to explain the atypical perceptual processing observed in autism. One account proposed by Pellicano and Burr (2012) to explain sensory integration difficulties in ASD, including perception, takes a Bayesian approach. In typical individuals, Bayesian models of perception propose that the perceptual system generates the most probable interpretation of the world around us by combining sensory information with prior expectations, underlying biases which shape overall perception. Pellicano and Burr (2012) argue that individuals with ASD are less influenced by prior expectations due to having weaker or flattened “perceptual priors” meaning that previous experience has less influence on perception. This theory struggles to explain why sometimes individuals with autism show “selective fusion”: the integration of sensory information in some conditions but not others.

An alternative framework called Enhanced Perceptual Functioning theory [EPF, Mottron and Burack (2001); Mottron et al. (2006)] is perhaps better able to account for the flexible integration of visual information observed in the literature by those with ASD. EPF theory specifies that lower level (sensory) processing in ASD is enhanced, which results in a reduced influence of higher level (or top–down) processes allowing for greater flexibility when integrating visual information. If higher-level visual information is not advantageous in completing a task, then it will not be integrated by those with ASD (unlike typically developing individuals), whereas if it is necessary then it will be integrated. One area of visual processing that could prove valuable in testing these theories is the combination of different cues (or sources of information) to depth.

## Binocular Disparity and Other Cues to Depth

While an overall perception of depth is elicited via combination of a number of different cues, one cue in particular, binocular disparity, has been shown to produce a strong sensation of depth even when other, highly reliable cues to depth are present (Hillis et al. 2004; Johnston et al. 1993; Knill and Saunders 2003; Lovell et al. 2012; Vuong et al. 2006). Binocular disparity occurs due to the eyes’ horizontal separation, causing each to register a slightly different view of the world. The brain exploits these differences, or disparities, in order to retrieve a three-dimensional layout of our surroundings.

The sensation of depth elicited by binocular disparity is termed stereopsis, and stereoscopic acuity (‘stereoacuity’) exhibits a large amount of individual variation in the ASD population, with a higher proportion of those with ASD being less sensitive to binocular disparity (Scharre and Creedon 1992; Adams et al. 2010; Anketell et al. 2013; Coulter et al. 2013; Black et al. 2013), In addition, there are also higher rates of strabismus in the ASD population (Kaplan et al. 1999; Scharre and Creedon 1992; Milne et al. 2009) which can impair binocular vision. While stereopsis is not the only cue to depth, its contribution to the overall percept of depth is notable (Saladin 1998); and it is arguable that impairment in the ability to utilise this cue results in a qualitatively different depth experience which can affect judgement of space (Barry 2009).

Accurate judgement of depth relies on the processing and integration of multiple cues including binocular disparity (mentioned above) but also information from texture and occlusion (Howard and Rogers 2012). The perception of texture patterns varies systematically with depth and thus can be used to estimate distance. Occlusion (discussed below), where one object is in front of another, puts strict limits on depth ordering.

## Cue Combination for Depth Perception in Autism

A recent study conducted by Bedford (2016) investigated how young people with autism or who were typically-developing integrated binocular disparity and texture gradient information. They did this by asking them to judge whether two surfaces that were tilted away from the observer had the same or different degree of slant. The cues to depth-defined slant were presented either alone or together; when both cues were available they could be either congruent (in agreement, specifying the same amount of slant) or conflicting (specifying different amounts of slant).

Bedford et al. (2016) found that typically-developing adolescents’ ability to judge slant was improved when both cues were available and they were congruent, compared to judgement of depth-defined slant using either cue alone. This benefit of combining sensory estimates is thought to be due to a reduction in uncertainty due to averaging (Hillis et al. 2002, 2004). When the cues were again presented together but were conflicting and signalled different slants, this reduced the typically-developing adolescents’ precision. This is because they could not help but average the cues, even when this made them worse at the task (determining depth-defined slant)—this deleterious effect is known as ‘mandatory fusion’ (Hillis et al. 2002).

Those with an ASD were able to integrate disparity and texture cues when congruent (showing increased judgement precision), but unlike their typical peers, they did not show the effects of mandatory fusion. That is, when cues were conflicting, the thresholds of those with ASD were identical to those obtained for their single best cue. Bedford et al. (2016) termed this ‘selective fusion’. The authors concluded that perception in individuals with ASD was more flexible and less governed by top-down feedback (see also Smith et al. 2015).

## Individual Differences in Stereoacuity

Both Bedford et al. (2016) and studies of typical depth cue integration (Hillis et al. 2002, 2004; Johnston et al. 1993; Knill and Saunders 2003; Landy et al. 1995) either explicitly state that their observers passed a clinical stereotest or state more generally that participants had no known stereo-vision problems/performed sufficiently on experimental tasks involving the use of stereo-vision for depth judgements. However, individual differences are abundant in depth perception research (Hillis et al. 2002; Knill and Saunders 2003; McKee 1983), and it has been suggested that the combination and relative weighting of cues may be largely observer-dependent (Nefs et al. 2010; Zalevski et al. 2007).

Though there is an emerging interest in the role of such individual differences in how depth cues are utilised and combined (Wilmer 2008), and it has been hypothesised that those with poor stereopsis may over-weight monocular depth cues (Hahn et al. 2010), the ramifications of differences in stereoacuity have not yet been explored. This is especially important in the case of developmental disorders such as ASD who are more likely to have poor binocular fusion and worse stereoacuity due to an increased prevalence in vision disorders (Denis et al. 1997; Kaplan et al. 1999; Milne et al. 2009; Scharre and Creedon 1992). It may be that the ‘selective fusion’ observed in ASD (Bedford et al. 2016) is an effect of limited stereopsis rather than a consequence of autism specific symptoms such as reduced top-down processing (Happé and Frith 2006; Mottron et al. 2006).

## Occlusion: A Qualitatively Different Depth Cue

Ordinal cues to depth, such as occlusion, are well-suited but under-used to assess how multiple cues are integrated into a final overall percept. Occlusion occurs when one object fully or partially hides another from view. The presence of occlusion has been demonstrated to hasten the processing of disparity and improve accuracy of depth judgements (Gillam and Borsting 1988). Moreover, when occlusion conflicts with other depth cues there is reduced reliance on disparity (Braunstein et al. 1986), and it can interfere with perception of depth overall (Schriever 1924; Stevenson and Körding 2009). The importance of the structural inference that occlusion affords have been noted previously (Harris and Wilcox 2009; Nakayama and Shimojo 1990; Tsai and Victor 2000); it is thought to aid in the perception of depth by providing hard constraints, where certain depth configurations are ruled out (Nakayama and Shimojo 1992).

The constraints provided by occlusion can be thought of as a type of perceptual prior, biasing overall perception away from an unlikely 3D layout. The current study aims to further explore visual cue integration in autism using psychophysical methods, as well as better characterise possible underlying causes of ‘selective fusion’ as observed by Bedford et al. (2016). If there is indeed a weaker influence of priors upon overall percept (Pellicano and Burr 2012), those with ASD would be expected to experience an attenuated effect of occlusion (see Hodgson and McGonigle-Chalmers 2011).

On the other hand, it has previously been shown that reliance on occlusion is especially strong in those with poor stereopsis (Braunstein et al. 1986). Therefore, if differences in cue integration in autism are caused by a reduction in the ability to utilise a single cue (such as binocular disparity) rather than a true difference in how the cues are integrated, it is predicted that ability to judge depth will be reduced to a greater degree for all individuals (i.e. ASD and non-ASD) with poor stereopsis compared to those with intact stereopsis when the occlusion cue conflicts with disparity information.

## The Current Study

In the present study, we experimentally assessed the effect of occlusion information, which is conflicting or congruent with disparity information, on the precision of relative disparity thresholds in adolescents and adults who are TD or have an ASD. In order to disambiguate whether any observed effects are due to occlusion, or simply use of the occluded figure as an additional source of disparity, control trials using a non-occluded reference figure were also included. Participants across both groups presented with a range of stereoscopic ability. Both crossed and uncrossed disparity threshold were estimated for all conditions.

There are three possible patterns of performance that could be shown

1. Occlusion is used as a prior, disparity thresholds and reaction times are reduced when occlusion is congruent and increase when it is conflicting. If the ASD group have flattened priors, or are influenced less by priors, then these effects will be reduced;
2. If the ASD group are able to flexibly combine cues, the occluded object may be used both as a prior and as a reference plane (dependent on whether the depth order of the occluded object is congruent with or conflicts with the disparity cue; Petrov and Glennerster 2006), so thresholds and reaction times may be reduced whenever it is present, compared to TD participants, who would theoretically only show lower thresholds in conditions involving no cue conflict;
3. Inclusion of an occluded or reference figure has the same effect on reaction times and thresholds for both groups.

It is also predicted that, regardless of diagnostic group, presence of congruent occlusion will decrease threshold and reaction time for those with poorer stereoacuity.

## Method

### Participants

Twenty-seven adults and adolescents with ASD and 27 TD participants were recruited from colleges (participants with ASD were recruited from both mainstream programs and specialist autism provisions), universities and community contacts (such as local autism support groups) in the greater Nottinghamshire area. Participant numbers are typical for this type of study (Hall et al. 2010). Inclusion criteria for the typical group included no self-report of diagnosed developmental conditions.

All participants completed the Adult Autism Spectrum Quotient (AQ; Baron-Cohen et al. 2001) and those in the ASD group were administered the Autism Diagnostic Observation Schedule—Module 4 (ADOS; Lord et al. 2000). All participants with ASD except for two met the cut-off score on the ADOS, with 12 participants obtaining the ‘autism spectrum’ classification and a further 13 obtaining the more severe ‘autism’ classification. Of the ASD group, thirteen individuals did not reach the 32-point criterion on the AQ (as recommended by Baron-Cohen et al. 2001), but this number was reduced to 7 when using the 26-point threshold suggested by Woodbury-Smith et al. (2005).

Participants were excluded for the following reasons:

1. TD participants—Adult Autism Spectrum Quotient score greater than 32 (AQ; Baron-Cohen et al. 2001): four participants;
2. ASD participants—not scoring above threshold on either the AQ or the Adult Autism Spectrum Quotient (AQ; Baron-Cohen et al. 2001): one participant;
3. Any participant who failed to perform better than chance on the baseline task (when disparity was set at 2.5 × threshold); 5 ASD [one of whom also met exclusion criteria (2)].

These exclusions left data for 22 ASD and 23 TD participants; their demographics are reported in Table 1.

**Table 1:** Participant characteristics

Measures	ASD	TD	t value	Significance
N	22	23		
Age (years)			−1.031	p = 0.309
Mean (SD)	21.8 (4.81)	23.6 (6.99)		
Range	16–34	16–40		
WASI verbal subscale (standardised)			−1.565	p = 0.128
Mean (SD)	96.8 (22.3)	106 (13.3)		
Range	61–131	87–130		
WASI performance subscale (standardised)			−1.138	p = 0.263
Mean (SD)	108 (17.4)	114 (14.1)		
Range	63–132	90–139		
WASI full scale (standardised)			−1.543	p = 0.134
Mean (SD)	104 (19.89)	111 (9.65)		
Range	65–125	92–133		
AQ			5.33	p < 0.001
Mean (SD)	31 (8.33)	19.1 (6.01)		
Range	10–45	10–30		
ADOS communication				
Mean (SD)	3.09 (1.11)	–		
Range	2–6	–		
ADOS social interaction				
Mean (SD)	6.91 (1.95)	–		
Range	3–11	–		

Comparisons of the typically-developed and autism spectrum disorder groups were performed using Student’s *t* test (two-tailed). While the WASI scores reported in the table are standardised, the raw scores were used in the analysis. *ADOS* autism diagnostic observation schedule, *ASD* autism spectrum disorder, *AQ adult autism-spectrum quotient, WASI* wechsler abbreviated scale of intelligence, *TD* typically-developing, *SD* standard deviation

All participants had normal or corrected-to-normal vision, but presence of stereopsis was not required. An initial measure of crossed stereoacuity was obtained using the TNO stereoacuity test (18th edition; *Nederlandse Organisatie voor* T*oegepast* N*atuurwetenschappelijk* O*nderzoek*; TNO, http://www.ootech.nl/).

## Apparatus

The stimuli were generated and presented using MATLAB, with the Psychophysics Toolbox (Brainard 1997; Kleiner et al. 2007; Pelli 1997) and Palamedes (Prins and Kingdom 2009) software packages and displayed on a Sony Viewsonic P225f CRT monitor calibrated using a PR655 spectro-photometer (Photo Research, Chatsworth, CA, USA). 3DPixx liquid–crystal shutter glasses controlled by a DataPixx (VPixx Technologies Inc˙, Saint-Bruno, QC, Canada) allowed for stereoscopic viewing. The monitor had a frame rate of 120 Hz (60 Hz with shutter glasses). The refresh rate of both the monitor and shutter glasses was confirmed using a photodiode and a Tektronix 2115 60 MHz oscilloscope. Viewing distance was 928 cm. One pixel subtended ∼ 0.00231° of visual angle (8.32 arc seconds).

## Stimuli and Design

The task was to indicate via a button-press which of two circles appeared closer in depth to the participant. The trial-wise dependent measures of interest included whether the participant perceived the comparison circle to be closer than the reference (see below), and the reaction time of their decision. For all trials, the targets (i.e. parts of the scene to be discriminated in depth) were bright circles (12.05 cd/m^2^) on a mid-luminance background (3.15 cd/m^2^). Only the red gun of the CRT monitor was used to display the stimuli.

**Fig. 1:**
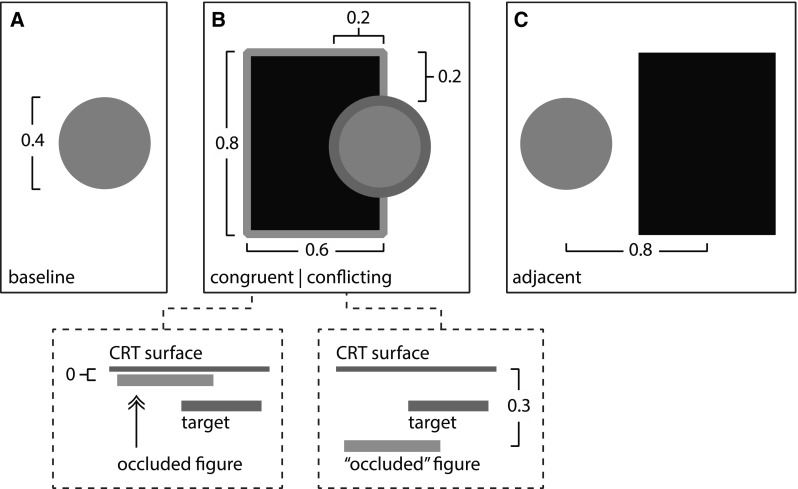
Examples of the different occlusion configurations. All measurements are in degrees. The task involved discriminating which of two *circle stimuli*—presented one above the other—was closer to the participant. The figure illustrates the position, in depth of one *circle* and the *rectangular* occluder. The *comparison circle* would be in front, or *behind this circle* (depending on the trial) **a** Baseline condition. **b** Conflicting occlusion and congruent occlusion. *Circle*(s) intruded upon a *rectangular* figure. In the congruent condition, the figure had zero disparity and always appeared to be *behind the circle*(s) in depth. For the conflict condition, the figure appeared in front of the circle in depth, though the *circle* still occluded the figure. The bird’s eye view shown in the *inset panels* depicts the crossed disparity cases; in the case of uncrossed disparities the stimuli all appeared ‘inside’ the CRT screen, and the disparities of the congruent and conflict figures were swapped. **c** Adjacent condition. The *circle* was presented to the *left of a rectangular* figure. (Color figure online)

The targets could be presented in four different occlusion configurations; baseline, congruent occlusion, conflicting occlusion, and figure-present-but-non occluding or ‘adjacent’ (Fig. 1). The baseline condition consisted of only the circle stimuli. Two circles were presented, one above the other—a reference circle of fixed disparity [0.16°] and a comparison circle, where the disparity was changed each trial. In the other three conditions a rectangle was also presented. This was low luminance (1.07 cd/ m^2^) and presented with zero disparity for crossed conditions, and 0.3° of disparity for uncrossed conditions (unless otherwise stated). In the congruent occlusion condition, the comparison and reference circles each occluded the rectangle. In the conflict condition, again the comparison and reference circles each occluded the rectangle, however, the disparity information provided by the rectangle indicated that it was in front of the circles (i.e. for crossed conditions, the rectangle was presented at a disparity of 0.3°, and at zero disparity for uncrossed conditions). Finally, in the ‘figure present but non-occluding’ or ‘adjacent’ condition, both the comparison and reference circles were presented beside the rectangle.

All occlusion configurations were presented in blocks of trials containing crossed or uncrossed disparities. For crossed disparities the stimulus viewed by the right eye was located further to the left, and vice versa for the left eye. Thus, the stimulus appeared to be floating in front of the surface of the CRT screen. For uncrossed disparities the stimulus viewed by the right eye was further to the right and vice versa for the left eye and the stimulus appeared to be inside the CRT screen.

## Procedure

The procedure was approved by the University of Nottingham’s School of Psychology Ethics Committee. Participants were seen within the School of Psychology in two or three sessions of 60–90 min. The experimental conditions were presented in separate blocks, with each block consisting of one condition. The order of presentation of conditions was counterbalanced between participants. The Weschler Abbreviated Scales of Intelligence (WASI) and Autism Diagnostic Observation Schedule (ADOS) were administered in separate sessions.

The laboratory-based experiment described here used standard psychophysical methods involving a 2-alternative-forced-choice paradigm (Pelli and Farell 1995, p. 29.9). In the experimental task, participants completed a relative-depth discrimination task where they had to choose which of two circles appeared closer to them. A trial consisted of a pair of stimuli (reference and comparison circles) presented one above the other for an unlimited amount of time until a response. Trials were separated by a random temporal jitter ranging between 0.5 and 1 s. A fixation cross with zero disparity remained in the centre of the screen at all times and participants were instructed to fixate on this cross throughout stimulus presentation. The task was composed of three stages: a combined demonstration and practice phase, a threshold estimation phase and a psychometric function estimation phase for each participant. This combined use of adaptive and constant methods allowed for efficient estimation of both threshold and slope parameter with relatively few trials and relatively novice participants.

### Demonstration and Practice Phase

The experimenter explained the task to the participants and showed four demonstration trials (one for each possible occlusion configuration). In all of these trials, the disparity of the comparison stimulus was set to 0.3°. Next, participants were presented with 20 practice trials, where the disparity of the stimulus was set adaptively, using a 2-down 1-up staircase (see next section). For the practice phase the starting value of the comparison stimulus was set at ±0.148° relative to the disparity of the reference stimulus. Participants indicated which stimulus appeared closer to them by pressing a key.

### Threshold Estimation Phase

A 2-down 1-up staircase with a 40-trial termination criterion was used to estimate the disparity at which the participant correctly identified the stimulus that was closer to them. This converges on a threshold of 80.35% correct. The comparison circle had a starting disparity value of ±0.074° relative to the disparity of the reference stimulus. Threshold was estimated as the mean of the last 4 reversals. The ratio of down and up stepsize (∆^−^/∆^+^) was set at 0.5488 (García-Pérez 1998). No feedback was given. A separate estimate of threshold was generated for each of the 8 conditions. The relative position of the reference and comparison stimuli (above or below the fixation cross) was randomised on each trial. A random amount of horizontal jitter was added to each stimulus to ensure that the task could not be completed monocularly.

### Psychometric Function Estimation Phase

A series of fixed disparity levels were then generated around the threshold obtained in the prior phase. The threshold was used as an anchor for generating five levels of disparity—one at the same level as the reference stimulus, 2 at the threshold level (one of which appeared in front of and one behind the reference stimulus) and 2 that were 2.5 times larger than the threshold (again, one that would appear in front of and one behind the reference stimulus). Each test disparity was presented 30 times. As in the previous phase, the relative vertical and horizontal position of the reference and comparison stimuli was randomised on each trial. Additionally, the presentation order of the comparison stimulus disparities was randomly shuffled before each block began.

## Data Analysis

### Psychometric Functions

All data were processed and analysed using quantitative parametric methods in R 3.2.2. The ratio of ‘target in front’ responses was calculated for each disparity level. These data were then fit with a psychometric function (as described in Wichmann and Hill 2001) for each participant condition-wise. This was done by fitting a Gaussian cumulative density function using ‘maximum likelihood estimation’ via R’s glm function (Knoblauch and Maloney 2012). An estimate of the threshold at which a participant specified the target as being in front of the reference 83.25% of trials and the slope of the psychometric function at this point were calculated using the threshold_slope function within the modelfree package. The thresholds were log-transformed to minimise the effects of skewness and kurtosis (Wesemann et al. 1987). The thresholds for the baseline or no-occlusion condition were used as covariates in the main analysis to assess the impact of stereoacuity. Thus occlusion configuration factor in the final analyses had three levels: congruent occlusion, conflicting occlusion and adjacent.

The disparity threshold and slope were modelled using mixed-effects linear regression (R package afex; Singmann and Bolker 2014). The models contained factors for occlusion configuration (occluding, conflict, adjacent) and disparity sign (crossed, uncrossed) of the stimuli, as well as diagnostic group (ASD, TD) and continuous predictors in the form of crossed and uncrossed stereoacuity (threshold obtained for the baseline occlusion configuration condition). The baseline threshold measures entered into the model as continuous predictors were also mean-centred to aid in interpretation of coefficient values. Sum contrast coding was used for categorical variables. Random intercepts and fully-crossed random slopes were included in the models (Barr et al. 2013). We report omnibus Type-III tests with denominator degrees of freedom, *F*-scaling factors, and *p* values obtained via Kenward-Roger approximation (Halekoh and Højsgaard 2014). All *p* values in the omnibus tests were Bonferroni-corrected (Dunn 1959, 1961). In cases where a significant main effect or interaction was found, pairwise comparisons were made with *p* values adjusted using the Tukey method (Tukey 1977), using the R package lsmeans (Lenth 2014).

### Reaction Times

Two RT outcome measures were calculated—median response speed (the reciprocal of RT), and the standard deviation of response speed (hereafter referred to as *response speed variability*). All RT measures were calculated separately for each combination of participant, condition, and disparity level. For both the median response speed and its standard deviation, the raw RT data were first trimmed so values fell between 250 ms and +2 standard deviations from the mean. The raw RT data were then transformed to speed by taking the reciprocal. Use of cut-offs and transformation when dealing with RT data is known to ameliorate the effect of slow outliers and thereby preserves power (Ratcliff 1993; Whelan 2008). Finally, both the psychometric parameters and RT outcome measures were screened for potential outliers with reference to the group. The threshold for rejection was set at 2.5 of the median absolute deviation (Leys et al. 2013).

Two linear mixed-effects models (using mixed function of the R package afex) were constructed. Like before, these models contained factors for occlusion configuration and disparity sign of the stimuli, as well as diagnostic group and continuous predictors in the form of crossed and uncrossed stereoscopic ability.

## Results

### Baseline Stereoacuity

Three measures of stereoacuity were obtained; the TNO test, and disparity threshold for the crossed and uncrossed baseline experimental conditions. Stereoacuity (across all three measures) ranged from 0.001° to 2.778°, with a median value of 0.017°. Bonferroni-corrected one-tailed t-tests showed that log-transformed stereoacuity scores differed significantly between groups for the *uncrossed* experimental baseline measure only [t(31.169) = − 1.754, p = 0.045]. Participants with ASD exhibited a worse average stereoacuity of −1.668 log10° [standard deviation (SD) = 0.516], whereas TD individuals had a better average stereoacuity of −1.885 log10° (SD = 0.267).

### Threshold

There were no main effects of or interaction including disparity sign (all *p* > 0.05), so all summary statistics for this measure are collapsed across disparity sign. There was no main effect of diagnostic group (p = 1.000), and there were no significant interactions.

**Fig. 2:**
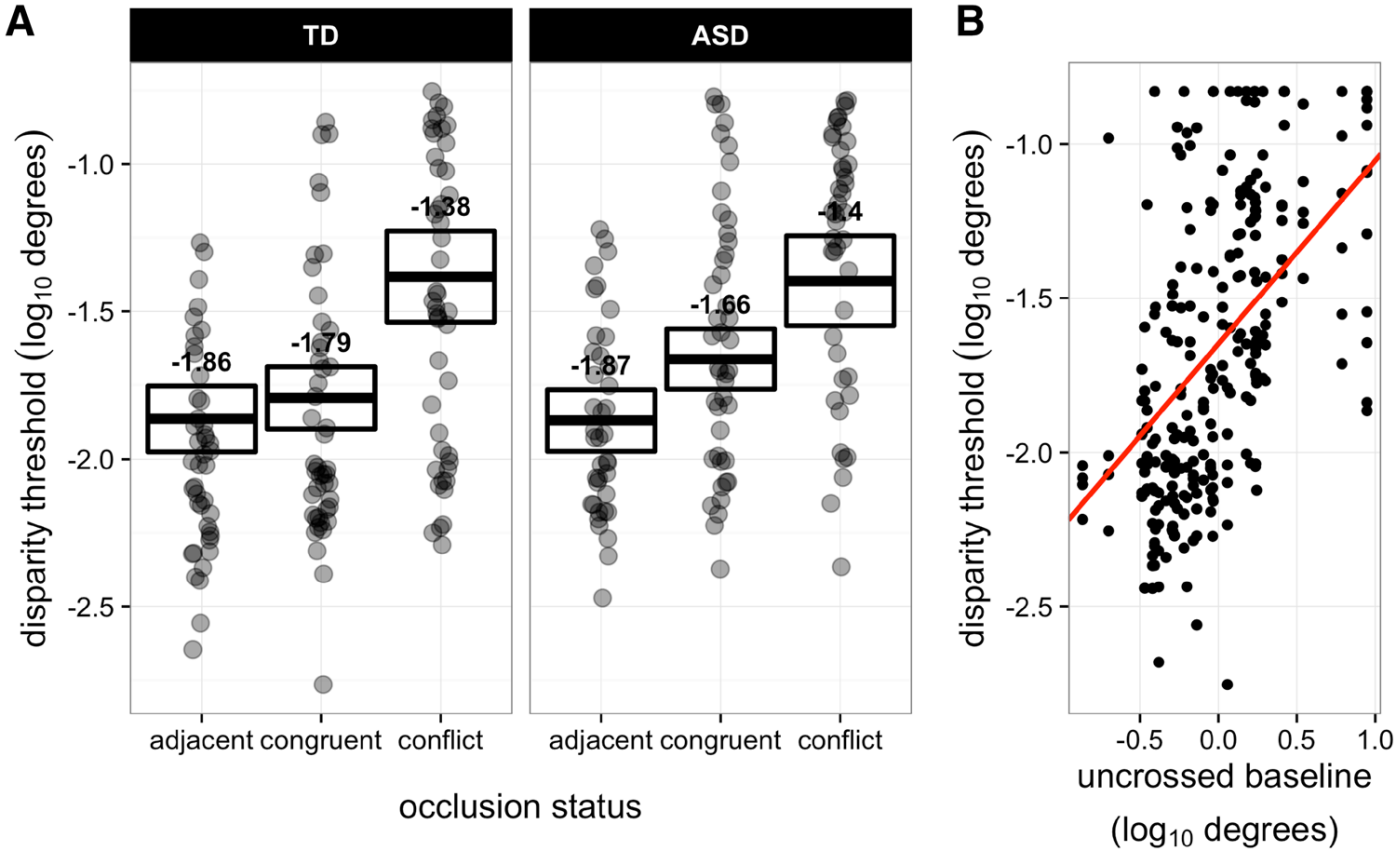
**a** Main effect of occlusion configuration and **b** uncrossed baseline stereoacuity on relative disparity threshold. **a** depicts least squares mean (±95% CI) disparity threshold for ASD and TD groups for the main effect of occlusion configuration, where the points are the data of individual observers. **b** shows disparity threshold as a function of uncrossed baseline stereoacuity; the points are the data of individual observers and the *red line* corresponds to the line-of-best-fit output by the linear mixed model. (Color figure online)

Occlusion configuration had a significant effect on threshold [F(2, 38.413) = 35.246, p < 0.001], see left panel of Fig. 2. Multiple pairwise comparisons revealed that the thresholds obtained from all three occlusion conditions significantly differed from one another. When the occlusion cue was conflicting, thresholds were higher compared to when the occlusion cue did not conflict [t(39.789) = −6.786, p  < 0.001], or when the figure was adjacent to the target [t(39.823) = −8.523, p < 0.001]. Thresholds were also significantly lower for the adjacent condition compared to the congruent occlusion condition [t(38.226) = −3.443, p = 0.004]. A significant main effect of uncrossed baseline stereoacuity [β = 0.74; F(1, 39.329) = 24.43, p < 0.001] showed that individuals with higher (i.e. worse) baseline uncrossed disparity thresholds were also likely to have increased thresholds for all other occlusion configurations.

### Slope

**Fig. 3:**
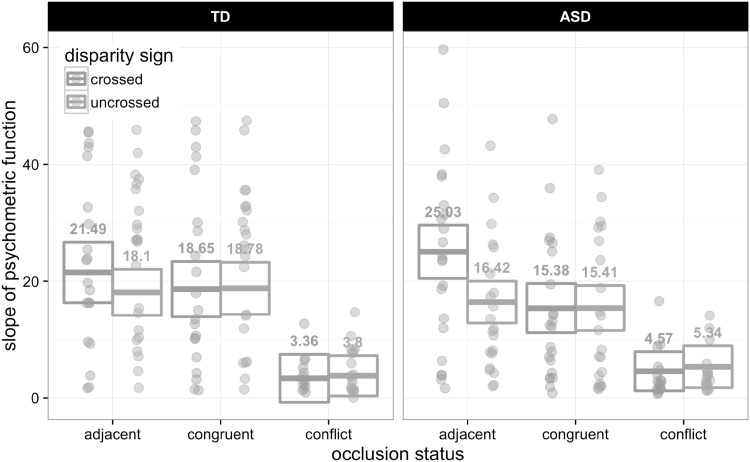
Mean (±95% CI) slope of psychometric function for ASD and TD groups for the two-way interaction between occlusion configuration and disparity sign. (Color figure online)

There was no significant main effect of, or interactions involving, diagnostic group (all *p* > 0.2), and there were no significant interactions. Similar main effects of occlusion configuration [F(2, 37.869) = 67.37, p < 0.001] and uncrossed baseline [F(1, 41.406) = 18.038, p < 0.001] measures also applied to the slope of the psychometric function (Fig. 3). The slope had a significantly shallower gradient in the case of conflicting occlusion [least squares mean (LSM) = 4.267 standard error of the mean (SE) = 0.878], compared to when the occlusion cue was congruent [LSM = 17.055 (SE = 1.299); t(37.558) = 8.905, p < 0.001], or when the figure was adjacent to the target [LSM = 20.261 (SE = 1.311); t(38.193) = 10.783, p < 0.001]. The slope was steeper for the adjacent condition than for the congruent occlusion condition [t(38.215) = 2.785, p = 0.022]. With regards to the baseline measures, there was an effect involving both crossed and uncrossed baseline stereoacuity, though the effect was stronger for uncrossed (β = −0.573) compared to crossed (β = −0.248).

For slope there was a significant interaction between disparity sign and occlusion configuration [F(2, 73.846) = 6.862, p = 0.007]. There was a difference in slope between crossed and uncrossed disparities only for the adjacent occlusion [t(93.584) = 3.782, p < 0.001], and not the congruent (p = 0.959) or conflicted types (p = 0.748).

### Reaction Time Measures

#### Response Speed

As observed for other measures, there was a main effect of occlusion configuration [F(2, 38.467) = 5.24, p = 0.039]. Response speed significantly differed between the conflict (LSM = 1.209 [SE = 0.04]) and congruent (LSM = 1.291 [SE = 0.042]) occlusion conditions [t(39.778) = 3.062, p = 0.011], and conflict and adjacent (LSM = 1.29 [SE = 0.044]) conditions[t(39.615) = 2.651, p = 0.03], but not for the congruent and adjacent conditions (p = 1). There was also a main effect of uncrossed baseline [F(1, 39.152) = 17.644, p < 0.001], where individuals with a higher uncrossed baseline threshold tended to respond faster (β = 0.973).

**Fig. 4:**
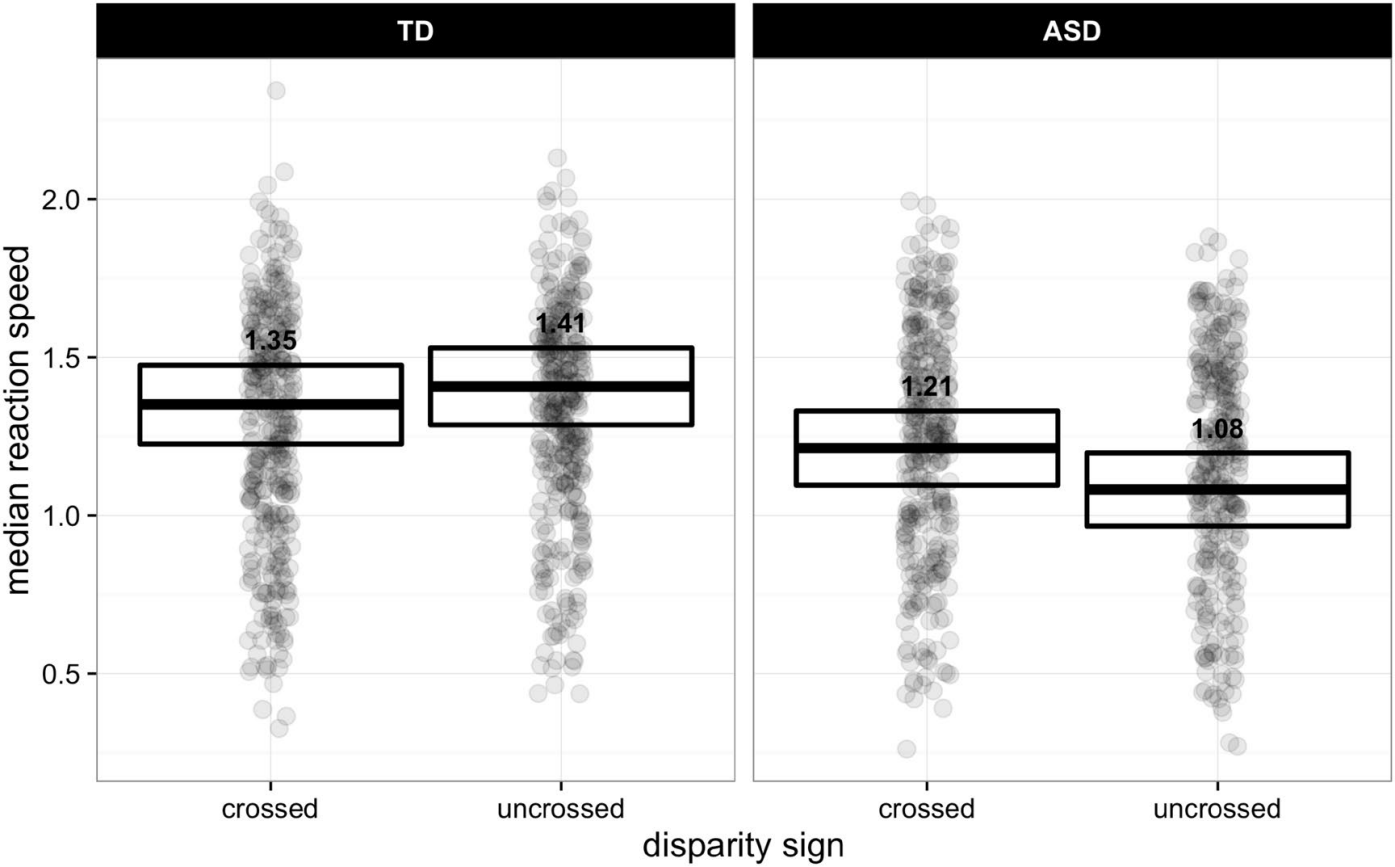
Mean (±95% CI) speed of response for ASD and TD groups for the two-way interaction between diagnostic group and disparity sign

Overall, participants with ASD exhibited slower response speeds (LSM = 1.147 [SE = 0.053]) than their TD counterparts [LSM = 1.379 (SE = 0.056)]; [F(1, 39.049) = 9.003, p = 0.019]. Though there was no main effect of disparity sign, there was an interaction between diagnostic group and disparity sign [F(1, 39.258) = 8.297, p = 0.026; which can be seen in Fig. 4]. Further analysis revealed that the ASD group had significantly slower response speeds to uncrossed disparities only [t(39.173) = 3.936, p < 0.001]; response speeds for crossed disparities did not differ between the two diagnostic groups (p = 0.112).

#### Response Speed Variability

Occlusion configuration also had an effect on response speed variability [F(2, 38.367) = 13.932, p < 0.001], with—as for median response speed—variability being significantly higher for the conflict condition (LSM = 0.415 [SE = 0.013]), compared to the congruent occlusion (LSM = 0.39 [SE = 0.011]; t(39.702) = −5.283, p < 0.001) or adjacent (LSM = 0.39 [SE = 0.011]; t(39.875) = −3.491, p = 0.003) conditions. Response speed variability did not significantly differ between the congruent occlusion and adjacent conditions (p = 0.134). However, an interaction between diagnostic group and occlusion configuration showed that this condition-dependent effect upon response speed variability was only present in the TD group; response speed variability did not significantly differ between occlusion conditions for the ASD group. Table 2 shows descriptives and pair-wise comparisons at all levels of diagnostic group and occlusion configuration.

**Table 2:** Interaction of diagnostic group and occlusion condition on response speed variability

Group	Condition	LSM	SE	Df	Lower CL	Upper CL	Posthoc
TD	Adjacent	0.397	0.016	39.125	0.365	0.429	a
	Occlusion	0.372	0.014	38.852	0.343	0.402	a
	Conflict	0.430	0.018	38.685	0.393	0.466	b
ASD	Adjacent	0.384	0.016	38.838	0.353	0.416	ab
	Occlusion	0.386	0.014	38.997	0.357	0.415	ab
	Conflict	0.401	0.018	40.120	0.365	0.438	ab

Pair-wise comparisons of least-squared means for two-way interaction between diagnostic group and occlusion condition, using Tukey’s honest significant difference test with α = 0.05. Rows containing the same letter are not significantly different to each other (Piepho 2004)

*TD* Typically-developing, *ASD* autism spectrum disorder, *LSM* least squares mean, *SE* standard error of the mean, *CL* confidence limit

Reported p values were adjusted for multiplicity using the Tukey method (Tukey 1977)

## Discussion

This study examined the impact of occlusion cues upon perceived depth in adults with and without ASD. Both groups performed as if they automatically integrated occlusion and disparity information to judge depth. In addition to this main finding there were interesting differences between the groups’ behaviour. Those with ASD were less sensitive to uncrossed disparities, measured psychophysically when no occlusion or reference planes were present (the ‘baseline’ occlusion configuration). Times taken to judge uncrossed relative disparities were increased for this group across all occlusion configurations. Variability of response speed did not depend on whether the occlusion cue was congruent for the ASD group, whereas response speed became more variable for the TD group when occlusion conflicted with disparity information. Additionally, across the TD and ASD groups, while an increase in relative disparity threshold for the baseline condition made it more likely that an individual would have similarly decreased performance across the occlusion and adjacent reference figure conditions, it was also related to faster response speeds.

### Group Differences

The main aim of the study was to determine whether individuals with autism exhibited true differences in depth cue utilisation, or if these were due to reduced stereopsis. There was a group difference in stereoacuity, with the ASD group exhibiting increased *uncrossed* thresholds compared to the TD group. Reduced stereoacuity in ASD has been identified previously (Adams et al. 2010; Anketell et al. 2013; Coulter et al. 2013; Scharre and Creedon 1992) but this is the first study to have tested for and observed a difference between crossed and uncrossed disparity threshold in ASD. An overall reduction in response speed (especially for uncrossed disparities) was also found. Slower reaction times are not characteristic of ASD (Ferraro 2016) so this may reflect a specific difficulty in processing disparity information.

A deficit localised to uncrossed disparities cannot be accounted for by the reported increase in prevalence of convergence insufficiency seen in autism (Milne et al. 2009; this would predict reduced sensitivity to crossed disparities). The perception of uncrossed disparities requires divergence, an ability that remains intact in ASD (Milne et al. 2009).

Uncrossed disparity refers to relative depth information further away than fixation. The effects of this on behaviour are unclear, since other cues to depth are also available. It is interesting to note that the ability to utilise uncrossed binocular disparities has been shown to reduce visual crowding (Astle et al. 2014), which improves the ability to recognise and respond appropriately to a stimulus when it is one of many possibly salient objects (Whitney and Levi 2011). It is possible that a reduced ability to make sense of crowded visual scenes could contribute to the reported sensations of being overwhelmed by sensory environments. This conclusion, however, is speculative at this stage.

It should be noted that those with autism exhibit a substantial increase in prevalence of strabismus compared to TD populations. Sensitivity to uncrossed disparity is specifically reduced in the case of exo-phoria or -tropia (Lam et al. 2002). While those with autism are not significantly more likely to have exo- compared to eso-deviations (Black et al. 2013; Denis et al. 1997; Ikeda et al. 2012; Kabatas et al. 2015; Kaplan et al. 1999; Milne et al. 2009; Scharre and Creedon 1992), the increase in prevalence of strabismus compared to the general population could contribute to this deficit.

Here we find evidence that populations with ASD do not differ from their TD peers in the utilisation of occlusion. Individuals with autism showed an identical pattern to their TD peers regarding the effect of occlusion configuration upon disparity threshold. The finding that those with ASD showed increased thresholds for conflicting compared to congruent occlusion indicates that the integration of metric and ordinal uni-modal cues is automatic in this population. This is inconsistent with the results of Bedford et al. (2016), who found that when two metric cues were incongruent, information from the cues was not necessarily combined in this group. While here we used a highly-reliable ordinal cue, Bedford et al. (2016) used texture. This use of qualitatively different cues may account for the discrepancy between our findings and those of Bedford et al. (2016).

It should be noted that in our study, where occlusion and disparity cues were conflicting those with ASD did not show increased response speed variability, unlike the TD group. This suggests that although the introduction of conflicting cues caused reduction in the ability to judge depth, it did not increase judgement uncertainty in the ASD group. This is not consistent with the flattened perceptual prior suggested by Pellicano and Burr (2012). A flattened occlusion prior could account for the fixed variability in response speed for those with ASD, but it does not readily explain the deleterious effect of conflicting occlusion on disparity threshold. A more parsimonious consideration could be that while the occlusion prior is similar for ASD and TD populations, there may be group differences in the perceptual decision-making aspect of the task used to assess the level of cue integration. In other words, though the performance of individuals with ASD may match the TD group, they do not experience the increase in difficulty experienced by the TD group.

### Limitations

The effects observed here were strong, the combination of a relatively small sample size and the heterogeneity present in individuals with an ASD means that unbeknown to the authors, there may be subgroups who were performing differently. Additionally, it is not known whether the automatic cue combination seen here in those with ASD is specific to the disparity and occlusion cues, or if this effect is more generalizable. Further studies using different cues are needed, especially in the case of cross-modal cue integration, where the presence of differences in ASD appears specific to a subset of the senses (Haigh et al. 2016; Hames et al. 2016).

## Conclusions

The present study examined the impact of occlusion cues upon perceived depth-from- disparity. Cue integration did not depend upon level of stereoacuity or autism diagnosis. Unlike previous work, people with ASD were found to automatically integrate conflicting depth cues, lending support to the idea that the occlusion prior remains intact in ASD and that the outcome of cue integration is similar to those who are TD. However, the decision-making undertaken to reach this final percept may differ between the ASD and TD groups. Future work should explore whether cue-specific likelihood distributions differ in autism, and if so, whether these are specific to those with ASD or are simply a function of a reduction in stereoacuity.
